# Analysis of Predictors Affecting Biomechanical Function of the Knee Joint and Its Relation to Anterior Knee Pain

**DOI:** 10.7759/cureus.21305

**Published:** 2022-01-16

**Authors:** Saurabh Chaudhary, Sanjeev K Jain, Nidhi Sharma, Supriti Bhatnagar

**Affiliations:** 1 Anatomy, Teerthanker Mahaveer Medical College and Research Center, Moradabad, IND

**Keywords:** sportsperson, q angle, knee joint, condylar distance, anterior knee pain

## Abstract

Background: Anterior knee pain is the most common problem in the young and sporting population. Quadriceps femoris angle and condylar distance are tools to assess the bio-mechanical function of the knee joint. The aim of this research was to give comparative data of quadriceps femoris angle and condylar distance in the Indian population (sedentary/sportsperson). The study also aims to know which parameter (condylar distance/quadriceps angle) is the better predictor for knee pain in the young Indian population.

Materials and methods: This study was composed of a total of 130 individuals suffering from anterior knee pain which was divided into two categories; Sedentary and sportsperson. Each category consisted of 65 individuals. Q angle (goniometric method) and condylar distance (manual caliper) of each participant were calculated. A comparison of body parameters was done by independent t-test. Comparison between the two parameters (condylar distance and quadriceps angle) was done to know which is the better predictor of anterior knee pain.

Results: Statistically significant sexual variation (p<0.05) was observed in both quadriceps angle and condylar distance in sedentary and sportsperson groups. Females had a higher value of Q angle than males (p<0.05). The difference in quadriceps angle was statistically significant (p<0.05) between sedentary and sportsperson groups. Cohen’s kappa coefficient of Q angle was 0.72 while that of bi-condylar distance was 0.49.

Conclusion: Q angle is a better indicator for anterior knee pain than condylar distance. Females in either category; sedentary and sportsperson, had higher Q angle in comparison to males making them more susceptible to disorders of the patellofemoral joint. Hence, encouragement and awareness are needed not only to carry out periodic screening of the susceptible population but also to emphasize its usage in clinical practice and the prognosis of the affected individual after treatment.

## Introduction

Anterior knee pain is defined as the pain and tenderness around the patellar region. The etiology is multifactorial and the underlying factors include patellar shape anomalies, muscular disproportions/weakness which finally leads to knee malalignment on movement of joint. The major cause behind this condition includes overuse injuries like tendinopathy, unstable patellar disorder, chondral and osteochondral degeneration, etc. Various physical activities like squatting, running, climbing stairs, and sitting crossed leg can exacerbate the conditions [1]. According to the recent literature, this condition is becoming a major problem in the young and sporting population [1-3]. The patellofemoral joint has been the topic of fascination for researchers for decades because individuals of all ages are affected by it. It is a complex joint having several contact points and multiple muscles, tissues, and ligaments attached to the patella which indirectly affects the bio-mechanical function of the knee joint [3].

Condylar distance is the distance between the two most prominent palpable points on condyles of the femur. It is measured by using a manual caliper when a volunteer stands erect in anatomical position and then flex the dominant leg to 90 degrees at the knee joint [4]. There are studies which suggest that there is no statistically significant difference between measurements obtained with calipers and different radiological methods [3,5]. Thus this parameter can be considered as a good predictor for knee pain. Studies also recommend that there is a strong association between intercondylar width and knee osteoarthritis [1,6]. 

Quadriceps femoris angle of the knee is an acute angle reflecting the placement of quadriceps musculature in relation to the bony structures like pelvis, thigh bone, and shinbone present below it [5]. Quadriceps femoris angle is referred excessive when the vector drawn from the mid-point of the patella increases laterally and eventually potentiates disorders of the patellofemoral joint. The main factor governing this is the pattern of quadricep femoris musculature [6,7].

The values of quadriceps femoris angle documented by several researchers globally vary according to the population involved in the study, thus confirming racial variation [8,9]. However, the accepted normal quadriceps femoris angle ought to be between 12 to 20 degrees. Q angle of an individual is said to be abnormal if male and female has a value higher than 15˚ and 20˚ respectively [10].

Q angle is frequently measured by orthopedics and physiotherapists as it reflects pathomechanics and biomechanics of the patellofemoral joint [4]. The severity of some medical conditions is associated with values of quadriceps femoris angle like anterior knee pain, patellar overload syndrome, hyper-mobile knee joint, patellofemoral instability, and dislocation of the patella [11,12].

The association between quadriceps femoris angle and injuries of the lower limb in sportsperson and military personnel is well documented [10]. Thus, it can be used as a screening tool in the susceptible population, who are at greater risk of wear, tear, or injury to the patellofemoral joint.

A number of studies have been carried out globally aimed to analyze the biomechanics of the knee joint but very few studies have been carried out to observe predictability of factors like quadriceps femoris angle and condylar distance for knee pain. So, the purpose to conduct this study was to know the differences in values of Q angle and condylar distance in the Indian population (sedentary and sportsperson). The secondary aim of the study is to know which parameter (condylar distance and quadriceps angle) is the better predictor for knee pain in the young Indian population.

## Materials and methods

This study was composed of 130 individuals who were suffering from anterior knee pain. Here, 65 individuals were sedentary and 65 were sportspersons. Subjects were selected as per inclusion and exclusion criteria. The study includes all patients between 18-35 years old while the patients suffering from spinal or neurological injury, present knee injury like fracture or dislocation of patella, and diseases of the knee like osteoarthritis were excluded from the study [12]. Measurement was done after securing the approval from the Institutional Ethical Committee (Ref. No.-TMMC&RC/IEC/19-20/116) at Teerthanker Mahaveer Medical College and Research Centre. A proper informed consent form was obtained from the patients and was spread out before commencing the measurements. Additionally, a short presentation was given so that all the participants would be accustomed after noting their name, age, sex, course, and region. Determination of a person, whether he/she is a sportsperson or not was done according to the definition given by Maron BJ [13] where an individual who participates in a prepared team or sport that needs consistent competition against others as a central element, places a high quality of excellence and achievement and entails some form of systematic training is defined as a sportsperson. Q angle (goniometric method) and condylar distance (manual caliper) of each participant (sedentary and sportsperson) were calculated.

Measurement of quadriceps angle [4]

**Figure 1:**
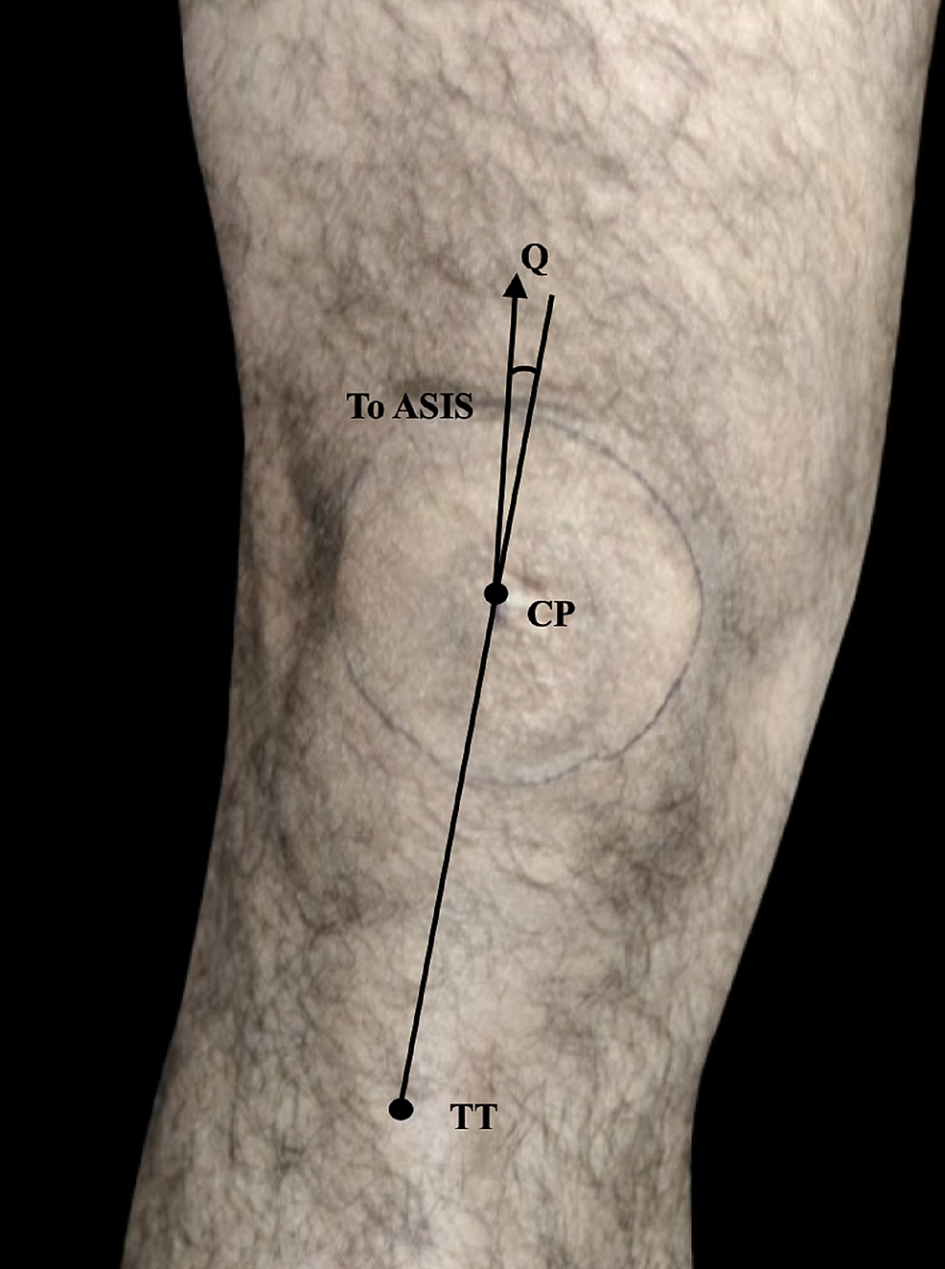
Measurement of Q angle of the left knee ASIS: anterior superior iliac spine; CP: centre of patella; TT: tibial tuberosity.

**Figure 2:**
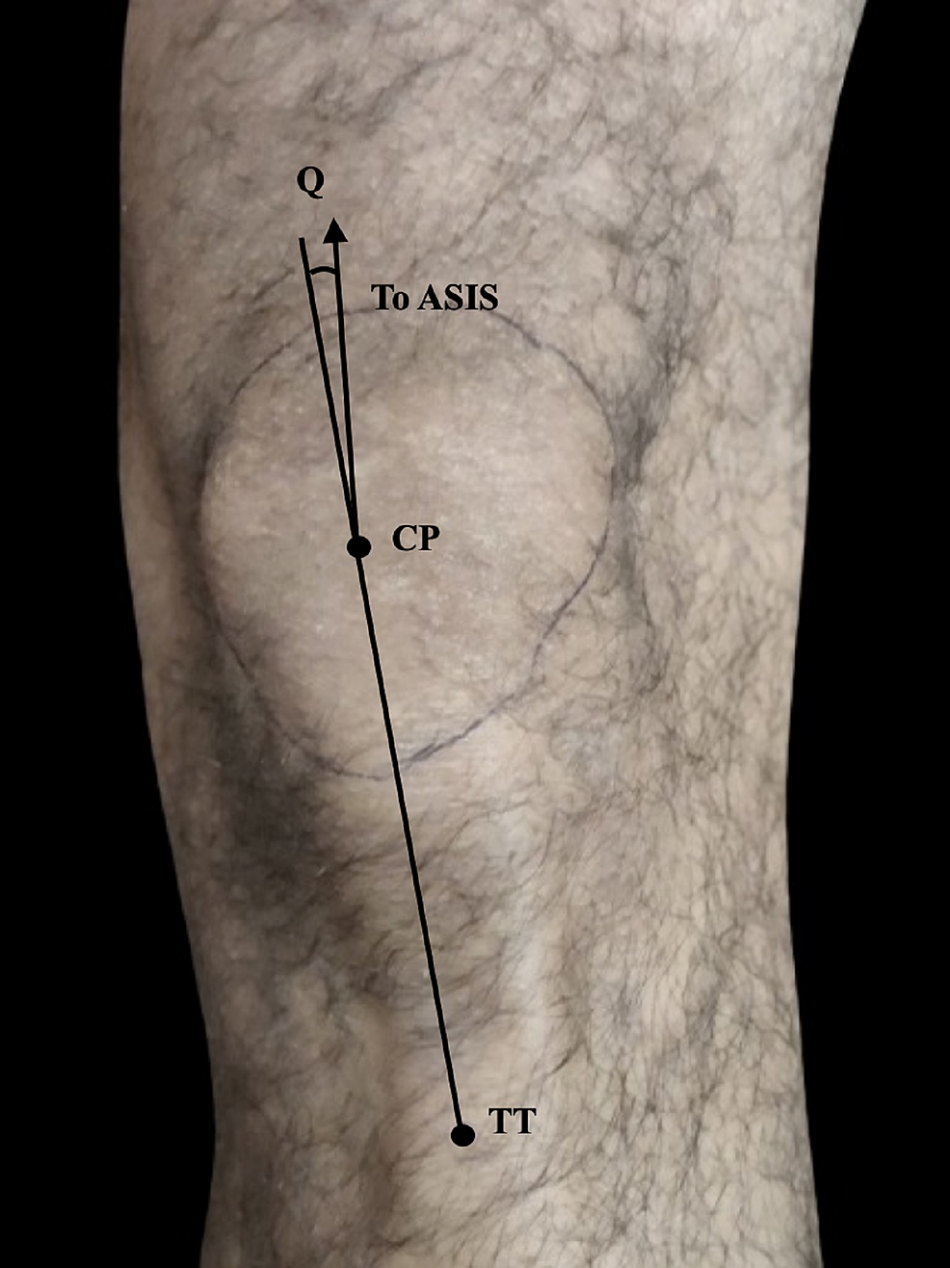
Measurement of Q angle of the right knee ASIS: anterior superior iliac spine; CP: centre of patella; TT: tibial tuberosity.

The goniometric method was adopted to calculate the quadriceps femoris angle. Firstly, participants were asked to be in a supine position followed by extension of leg and relaxation of quadriceps musculature. Then, participants were requested to put the feet in neutral rotation in such a way that toes were facing upward and feet is perpendicular with respect to the surface. Three bony points; the anterior superior iliac spine (ASIS), the center of tibial tuberosity (TT), and the centre of patella (CP) were identified and marked by a marker (Figures 1-2).

For identifying CP, the contour of patella was drawn after appreciating the borders without stretching skin. The CP was referred to as the point where maximum vertical diameter meets with maximum transverse diameter. The Centre of TT was the point having maximum appreciation. A measuring scale or tape was used to draw a straight line from ASIS to CP and another line from TT to CP.

The hinge of the goniometer (least count of the goniometer-1 degree) was placed at CP and the arm of the goniometer was arranged in such a way that one is positioned in the straight line drawn from ASIS to TT and another arm to the line from the ASIS to CP. The acute angle formed between the two arms of the goniometer was recorded as quadriceps femoris angle [4]. The normal range of quadriceps angle is 12-22 degrees [10].

Measurement of condylar distance [4]

A manual caliper (least count-1mm) was used to measure the femoral condylar distance of each participant. Firstly, participants were asked to stand erect in anatomical position and then flex the dominant leg to 90 degrees at the knee joint. Condyles of femur became prominent and can be easily appreciated. The manual caliper has two arms; fixed and moveable. The fixed arm was put on femoral’s lateral condyle followed by adjustment of the movable arm on the medial condyle. The distance covered was shown in cm in caliper which was recorded and written on pro forma sheet.

For statistical analysis, SPSS version 23 (IBM Corp., Armonk, NY) was used. A comparison of body parameters was done by independent t-test. The p value < 0.05 was considered statistically significant.

Cohen’s kappa (k) was calculated to measure the reliability of two parameters and thus identify which parameter (condylar distance/quadricep angle) is the better predictor for anterior knee pain. Interpretation of values of kappa coefficient in terms of agreement as follows: < 0 = No; 0.01-0.20 = None to modest; 0.21-0.40 = Reasonable; 0.41-0.60 = Moderate; 0.61-0.80 = Substantial & 0.81-1.00 = Almost ideal.

## Results

In this study, a total of 130 patients with anterior knee pain were included wherein 65 each was sedentary and sportsperson. The Q angle and condylar distance were measured in both categories. In addition to this, the correlation of quadriceps angle with that of condylar distance was evaluated in both sedentary and sportsperson groups. Cohen’s kappa (k) was calculated to measure the reliability of two parameters and thus identify which parameter (condylar distance/quadriceps angle) is the better predictor for anterior knee pain.

Table 1 depicts that no statistically significant difference (>0.05) in condylar distance is found between sedentary and sportsperson. The value of Q angle in sportsperson is lower as compared to sedentary which is statistically significant (p<0.05)

**Table 1:** Comparison of parameters between sedentary and sportsperson

S.N.	Parameters	Sedentary	Sportsperson	t-value	p-value
		Mean + S.D	Mean + S.D		
1	Condylar Distance (cm)	8.56 + 0.70	8.68 + 0.97	0.85	0.40
2	Q - angle (degree)	15.89 + 2.28	12.25 + 1.66	10.43	<0.05

Table 2 showed that the value of the condylar distance is statistically higher (p<0.05) in males as compared to females in the sedentary group. However, value of Q angle is statistically higher (p<0.05) in females as compared to males.

**Table 2:** Comparison of parameters between males and females in the sedentary group

S.N.	Parameters	Sedentary		t-value	p-value
		Male	Female		
		Mean + S.D	Mean + S.D		
1	Condylar Distance (cm)	9.08 + 0.39	7.95 + 0.44	10.10	<0.05
2	Q - angle (degree)	15.11 + 2.72	16.80 + 1.10	3.18	<0.05

Table 3 showed that the value of condylar distance is statistically higher (p<0.05) in males as compared to females in the sportsperson group. However, value of Q angle is statistically higher (p<0.05) in females as compared to males. These results were similar to the results in sedentary group

**Table 3:** Comparison of body parameters between males and females in the sportsperson group

S.N .	Parameters	Sportsperson		t-value	p-value
		Male	Female		
		Mean + S.D	Mean + S.D		
1	Condylar Distance (cm)	9.47 + 0.45	7.77 + 0.50	14.43	<0.05
2	Q- angle (degree)	11.46 + 1.46	13.17 + 1.40	4.80	<0.05

Cohen’s kappa of quadriceps angle for anterior knee pain

Out of 60 patients with anterior knee pain, 45 patients had abnormal Q angle and 12 had normal Q angle while in three patients, it was confirmed that either anterior knee pain was not the primary condition or the patient did not co-operate. The Cohen’s kappa coefficient calculated is 0.72 (Substantial).

Cohen’s kappa of condylar distance for anterior knee pain

Out of 60 patients with anterior knee pain, 41 patients had bi-condylar distance above the usual values in the Indian population and 12 had usual values while in seven patients, it was confirmed that anterior knee pain was not the primary condition or the patient did not co-operate. The Cohen’s kappa coefficient calculated was 0.49 (Moderate).

Cohen’s kappa coefficient of Q angle was 0.72 while that of bi-condylar distance was 0.49. Hence, Q angle is better indicator for anterior knee pain.

## Discussion

In this study, a comparison of Q angle and condylar distance were done in two groups (sedentary and sportsperson) as well as sexual dimorphism was also seen in both categories. In the present study, the Q angle of sedentary was found to be 15.89 + 2.28 cm and that of a sportsperson was 12.25 + 1.66 cm and the difference was statistically significant (p<0.05). Research conducted by Elioz M et al., [14] on quadriceps angle in sedentary, amateur athletes and professional athletes was 10, 9.01, and 8.72 respectively. Thus, similar to this study, lower value of Q angle was found in sportspersons as compared to the sedentary group. This variation in values of quadriceps angle in the two groups depends on the intensity and level of physical activity [15]. The sportsperson who are indulged in vigorous exercises had lower values as compared to those who have a sedentary lifestyle. Some studies further stated that there is an association between quadriceps angle and indexes such as length of the femur and pelvic length [15]. The value of the Q angle of sedentary was similar to the findings of Prakash SS et al., 2019 [16].

The average Q angle varies within range 8°-22.8° in the various populations [17,18]. Explanation to this variation could be factors such as ethnicity, sex, age, and height of participants included in the study. The body’s position and placement of foot along with the level of contraction of quadriceps musculature also greatly influence the quadriceps femoris angle [11].

While examining differences of Q angle between sedentary and sportsperson on the basis of gender, Q angle of the sedentary male was found to be 15.11 + 2.72 cm and that of female was 16.80 + 1.10 cm and the difference was statistically significant (p<0.05). The average value obtained here is approximately the same as the values observed by Daneshmandi [19]. Meanwhile, in sportsperson Q angle of males was 11.46 + 1.46 cm and that of females was 13.17 + 1.39 cm and variation was statistically significant (p<0.05). In this study, we can see that the average quadriceps angle in the female population was also majorly greater in comparison to the male population. Here goniometer was used to approximate the Q angle and on the basis of gender, the difference was observed to be 1.71 and 1.69 in sportsperson and sedentary respectively. However, the reasoning for this revelation is still not clear. The possible reasoning for the females showing a higher Q angle than that of males can be associated with the pelvis of females which is much wider in comparison to males and since the distance from the patella to the pelvis is longer than the distance from the TT to patella, it can be derived that placement of the ASIS has a greater effect on the Q angle [4]. On the contrary, to this explanation, Jaiyesimi AO et al. pointed out that the variation in the values is associated with the height of the individual rather than the placement of bony landmarks i.e. since male participants tend to have a greater height than females, Q angle in males is lower in comparison to females [20]. Values of Q angle obtained in males and females in this study is greater than majority of the reported range [4,21]. Hence, population of India are at greater risk of having abnormalities in knee joint.

The peculiarity of this study is that, we have tried to find out which parameter (condylar distance/quadriceps angle) is the better predictor for knee pain. Different studies have proved that both quadriceps angle and condylar distance have strong correlation with the anterior knee pain [22]. For Q angle and condylar distance, the kappa coefficient is 0.72 (Substantial) and 0.49 (Moderate) respectively. Hence, Q angle is considered as better indicator for anterior knee pain.

Limitations

This study provides comparative statistics on the basis of gender and categorical variation. No follow up mechanism was placed for susceptible, borderline or risk group. Data from follow up could have alleviated our understanding of Q angle and its impact to new level regarding the time taken for symptoms to arise, role of physical activities, occupation or sports and much more. We were unable to get in hold of national and international players. Their inclusion could have helped us to better understand the effects of Q angle and condylar distance on sportsperson.

## Conclusions

Statistically significant (p<0.05) difference in Q angle was found between sedentary and sportsperson and as well as on the basis of gender in both categories. Females in both categories had a higher Q angle in comparison to males making them more susceptible to disorders of the patellofemoral joint. In addition, Cohen’s kappa coefficient of Q angle was 0.72 while that of bi-condylar distance was 0.49. This showed Q angle is a better indicator for anterior knee pain. Performance of lower extremity is extremely important in physical activity. Hence, finding out different body parameters that can influence the values of the Q angle of an individual is the need of the hour. It has greater significance to sportsperson, in particular females, who are involved in different competitive sports and physical activities. Therefore, encouragement and awareness are needed not only to carry out periodic screening of the susceptible population but also to emphasize its usage in clinical practice and the prognosis of the affected individual after treatment. Different correctional exercises can be started as a precaution to prevent future disorders. In individuals going through rehabilitation programs, a periodic checkup of the Q angle will provide vital info in evaluating the strategy of treatment and modifying it if necessary. These findings will create awareness among coaches and managers of sportspersons as well as in the overall population.
